# Genetic services survey—experience of people with rare diseases and their families accessing genetic services in the Irish Republic

**DOI:** 10.1007/s12687-023-00664-w

**Published:** 2023-08-26

**Authors:** AJ Ward, DM Lambert, D Butterly, JJ O’Byrne, V McGrath, SA Lynch

**Affiliations:** 1 University College Dublin, School of Medicine, Dublin, Ireland; 2 Mater Misericordiae University Hospital, National Centre for Inherited Metabolic Disorders, Dublin, Ireland; 3 Trinity College Dublin, School of Medicine, Dublin, Ireland; 4 Rare Diseases Ireland, Dublin, Ireland; 5 Children’s Health Ireland (CHI) at Crumlin, Clinical Genetics, Dublin, Ireland

**Keywords:** Genetic services, Genetic testing, Patient experience, Genomic mainstreaming

## Abstract

Irish Health Service objectives state that patients with rare diseases should have timely access to genomic diagnostics with appropriate pre and post-test counselling. However, waiting times for clinical genetics outpatient appointments, during the study period, were up to two years as staffing levels remain low. A targeted public online survey was conducted in January 2022 to capture the experiences of Rare Disease families trying to access genetic testing and clinical genetic clinics in the Irish Republic. Irish patients experience significant waiting times to access clinical genetic services and self-report anxiety and stress, related to delayed access to diagnosis, clarity around recurrence risk and follow-up management. This negatively impacts personal decisions around family planning, education and employment and has a significant impact on family members seeking clarity on their own risk. Mainstream genetic testing activity is significant. Families report concern over the competency of health care professionals arranging and delivering genetic results and delays in accessing clinical genetics expertise to take them through the clinical implications. Timely access to clinical genetics expertise is important to ensure families with rare diseases have an appropriate understanding of the medical and reproductive implications of a genetic diagnosis and access to relevant care pathways. A national framework to develop competency in genomic literacy for health-care professionals including a national genetic test directory may be beneficial. Clinical genetics teams require ongoing support and investment to ensure the delivery of a safe and effective service for Irish families with rare diseases.

**Supplementary Information:**

The online version contains supplementary material available at 10.1007/s12687-023-00664-w.

## Background

National waiting times for clinical genetics outpatient appointments in the Republic of Ireland (ROI) are up to two years as staffing levels, compared to international standards, have remained a challenge (Lynch and Borg 2016; Bradley and Lynch 2021; Walsh et al. 2022). National Treatment Purchase Fund (2022) data shows that in December 2022 there were 2799 patients waiting for clinical genetics outpatient appointment with 35.9% (1002/2799) waiting over 12 months.

There is a single clinical genetic service in the ROI, based in Dublin, which accepts paediatric, fetal medicine and adult referrals from all regions of the Irish Republic and covers general and cancer cases. This clinical genetic service is based in a paediatric hospital with no ring-fenced budget. There is a separate Dublin-based adult cancer genetic service led by two oncologists and a single Clinical Geneticist provides a perinatal genomic medicine service in one Dublin Obstetric hospital. At the time of the study, there were 5 full-time clinical geneticists based in Dublin providing nationwide service to a population of 5 million.

Some European Union countries have recommended waiting times for outpatient appointments and national gatekeeping structures such as genomic testing directories to guide clinicians (National Health Service NHS England 2023). No such national frameworks exist in Ireland and often genomic testing is accessed via overseas commercial companies in a fragmented manner.

The Irish Health Service Executive (2019) (HSE) Model of Care for Rare Diseases recommends that all people with rare diseases have timely access to genomic diagnostics nationally and appropriate pre- and post-test counselling or a cross-border care reimbursement pathway. The Health Service Executive (2022) National Strategy for Accelerating Genetic and Genomic Medicine in Ireland aims to set out key national objectives in strengthening and developing the clinical and laboratory Genetic workforce needed to deliver timely access to genomic care.

With the rapid development of affordable genomic testing in the context of long wait times for clinical genetic services, ad hoc mainstreaming activity within the Irish healthcare system has grown to fill these gaps. We wanted to consider access to genetic testing and clinical genetics expertise separately as, in Ireland, a significant proportion of genetic testing is carried out by mainstream clinical services. Our survey aimed to establish evidence of mainstream activity and patients’ experience of this.

Long waiting times for clinical genetic services can have potentially significant health consequences due to delayed access to management and the risk of patients or key relatives dying with a potential impact on the ability of healthcare professionals to clarify the diagnosis, recurrence risk and wider implications for the family (Bradley and Lynch 2021). Financial waste due to duplicate referrals has also been highlighted (Walsh et al. 2022).

However, limited literature exists on the psychosocial impact of long waits on patient and family well-being, decision-making around personal relationships, family planning, education and employment or the possible wider impact for extended relatives who are seeking clarity about their own or their children’s risk of a familial condition.

### Aims

This study aimed to (1) identify the personal impact of long waits on families, (2) understand families’ experiences when trying to access Irish clinical genetics services, (3) explore the experience of receiving genetic testing through non-genetic healthcare teams in the absence of a national mainstreaming framework, and (4) assess public awareness of the role of genetic counsellors.

## Methods

### Survey development

The survey (see supplementary information Figure 1) was collaboratively written with Rare Diseases Ireland (https://rdi.ie/), the Irish national rare disease patient organization alliance, in accordance with quality guidelines for Internet surveys of the CHERRIES quality checklist (Eysenbach 2004). This targeted public online survey was aimed at people with rare diseases and their family members to solicit their views on genetic testing and genetic counselling in Ireland.

The study was approved by the Children’s Health Ireland (CHI) Research Ethics Committee (GEN/937/21). A data protection impact assessment was undertaken and approved by the Data Protection Officer at CHI-Crumlin. People aged 18 years or older living with a rare condition or family members of people with rare conditions were considered eligible participants. Survey logic was applied to gatekeeping questions to direct respondents to relevant sections.

Dissemination was via patient advocacy partner Rare Diseases Ireland social media channels—their website and Twitter account were used to promote the survey and disseminate the links. There was a final promotion at a public Rare Disease Day event on the 16^th^ of February 2022. The survey was open from 03/01/2022 to 21/02/2022.

Responses received were checked for the level of completion. Those responses with a low completion level or time spent of less than 5 minutes were removed. Internet protocol (IP) address analysis was used to ensure that single users did not respond multiple times except where a person with a rare disease and their family member were giving separate responses from the same device. Data was cleaned to remove any potentially identifiable information, e.g. family or clinician names. Rare condition names were replaced by higher level categories, e.g. chromosomal condition, neurodevelopmental disorder. Any responses from Northern Ireland were excluded as it has a separate jurisdiction and health service system. Excel was utilized for the univariate descriptive analysis of the data. The graphical and statistical functions of SPSS and an online Z-test calculator were employed for quantitative data analysis.

In 7 questions across the survey respondents were given an opportunity to describe their experiences of accessing clinical genetics services using free-text boxes. Quotes were redacted from those who did not give permission for usage. Two researchers, one with a patient advocacy background and the other a genetic counsellor (GC), independently reviewed the free-text comments and identified and agreed recurrent overarching themes. A third researcher independently quantified all comments relating to these. Key quotes were selected which highlighted these themes.

## Results

### Respondents

There were 204 survey responses, of which 171 were from eligible participants. Responses were obtained from all 9 of the Irish Community Healthcare Organization (CHO) regions in the Republic of Ireland with 36.2% from the greater Dublin area and over 4.7% from each of the other regions. Respondents were predominantly female (94.2%, 162/171), white Irish (93%) and the family member / carer of a person living with a rare condition, answering on their behalf (67.8%). Respondents were asked to describe the age and diagnosis status of the person living with the rare condition. In 49.7% of responses, the person with the rare condition was a child ≤ 15 years and 73.5% had a named rare condition. Full demographic information is outlined in Table 1.

**Table 1:** Demographic characteristics of respondents completing the survey and details of the person living with the rare condition

*Demographics*		*N*	*%*
Gender (*n*=171)	Male	9	5.3%
	Female	161	94.2%
Age of person with the rare condition (*n*=171)	≤ 15 years	85	49.7%
	16–50 years	71	41.5%
	˃ 50 years	12	7.0%
Ethnicity (*n*=171)	White Irish	159	93.0%
	White non-Irish	12	7.0%
Respondents (*n=*171)	Living with a rare condition accessing genetic services for themselves	32	18.7%
	Family member / carer of a person living with a rare condition and answering on their behalf	116	67.8%
	Family members of a person living with a rare condition accessing genetic services for themselves	19	11.1%
Disease status of person wth rare condition (*n*=170)	Have a named rare condition	125	73.5%
	Still seeking diagnosis	11	6.5%
	Syndrome without a name	7	4.1%

### Aspects of health affected

**Fig. 1:**
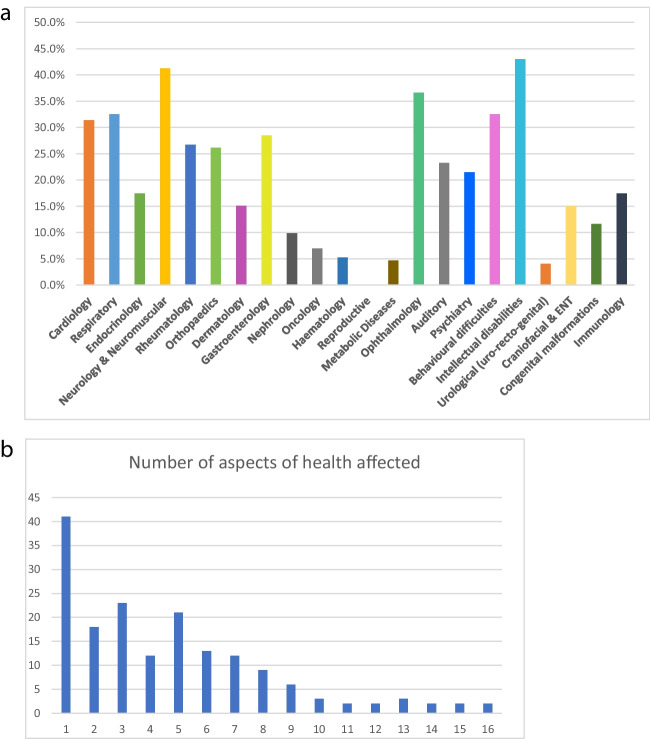
**a** Aspects of health affected. **b** Number of aspects of health affected

Survey participants were asked to indicate which of the 22 aspects of health are affected by the rare condition. The most common responses were 43% intellectual disability, 41.5% brain/ spinal cord / nerves, 36.8% vision/eyes, 32.8% lungs, 32.8% behavioral difficulties, 31.6% heart, and 21.6% mental health. Figure 1a outlines the health categories affected.

Survey respondents reported an average (mean) of 4.6 health areas affected (range 1–16) with 45% reporting 5 or more and 9% over 10 (Figure 1b).

### Experience in genetic testing and clinical genetic services

Univariate analysis of genetic testing questions is shown in Table 2. The majority of respondents had received genetic testing (94.7%, 162/171) with 71.5% (113/158) reporting that this had led to a diagnosis.

**Table 2:** Univariate results: Genetic testing

*Genetic testing*		*N*	*%*
Had genetic testing (*n*=171)	Yes	162	94.7%
	No	7	4.1%
Genetic testing led to a diagnosis (*n*=158):	Yes	113	71.5%
	No	25	15.8%
Genetic testing was arranged by (*n*=161):	Genetic consultant / genetic counsellor	38	23.6%
	GP/family doctor	9	5.6%
	Another consultant	114	70.8%
Was the Genetic test result explained by the same team who arranged testing (*n*=168)	Yes	102	60.7%
	No	42	25.0%
Professional that gave the genetic testing result instead (*n*=41)	Genetic consultant	13	31.7%
	Genetic counsellor	7	17.1%
	GP/family doctor	2	4.9%
	Another consultant	14	34.1%
	Unsure	5	12.2%
Wait time for genetic test results (*n*=161)	0–3 months	69	42.6%
	4-6 months	52	32.1%
	>6 months	38	23.5%
Communication method of genetic test results (*n*=166)	In-person appointment	103	62.0%
	Phone call	45	27.1%
	Letter	34	20.5%
	E-mail	5	3.0%
Preferred communication method of genetic test results (*n*=159)	In-person appointment	132	83.0%
	Phone call	32	20.1%
	Letter	17	10.7%
	Video call	10	6.3%
Satisfaction with genetic testing experience (*n*=168)	Very or quite satisfied	63	37.5%
	Neither satisfied nor dissatisfied	33	19.6%
	Very or quite dissatisfied	69	41.1%

Fifty-six per cent (90/159) of families waited 4 months or more for their genetic test results. Seventy-six per cent (123/161) had their genetic test arranged by a non-Genetic healthcare professional. The top 5 mainstreaming specialisms, accounting for 77% of all reported mainstreamed test requests, were neurologist (28%), paediatrician (24%), neonatologist (11%), cardiologist (10%), and metabolic specialist (4%). The full list is shown in supplemental Table 1. The 25% (42/168) of respondents who had their genetic test result explained by a different physician/team from the one who arranged the test, waited longer for full clinical interpretation of their test results (*z*=2.457; *p*<0.01). In 48.8% of these cases, a clinical genetics consultant or genetic counsellor explained the results.

Participants were asked how their genetic test results were communicated—this was predominantly by in-person appointment (62.0%) or phone call (27.1%). In-person appointment was strongly favoured for results communication (83%).

Respondents reported their satisfaction level with the genetic testing experience—37.5% indicated they were very or quite satisfied and 41.1% very or quite dissatisfied. Longer waiters (>6 months) were more likely to be dissatisfied with their experience of genetic testing (*z*=3.69; *p*<0.01).

Univariate data from clinical genetics appointment questions is shown in Table 3. Respondents were asked if they had been referred or attended a clinical genetics appointment with a genetic consultant or genetic counsellor. The majority had already attended (51.4%) or had been referred and were waiting for their appointment (12.2%). These were mainly public (79.2%) appointments with 19.2% accessing private sector services. Waiting times varied with 36.4% being seen within 6 months, 14% between 6 and 12 months and 30.8% waiting over 12 months. However, 49.6% waited over 12 months. A total of 18.2% (27/148) responded that they had not been referred to clinical genetics, but desired referral. When a diagnosis was made through attendance at the clinical genetics appointment higher levels of satisfaction were reported (*z=*3.87; *p*<0.001).

**Table 3:** Univariate results: Genetic Services questions

*Genetic services*		*N*	*%*
Referral status to genetic consultant / genetic counsellor consultation (*n*=148)	Yes - have already attended	76	51.4%
	Yes - waiting for the first appointment	18	12.2%
	No - have not been referred, but would like to be	27	18.2%
	No - have not been referred and do not want to be	8	5.4%
Type of genetic consultant / genetic counsellor appointment or referral (*n*=120)	Private	23	19.2%
	Public	95	79.2%
Wait time for genetic consultant / genetic counsellor appointment (n=143)	0-3 months	35	24.5%
	3-6 months	17	11.9%
	6-12 months	20	14.0%
	12-15 months	7	4.9%
	15-18 months	5	3.5%
	18-24 months	14	9.8%
	>2 years	18	12.6%

A total of 164/171 participants gave permission for their quotes in the free text sections to be shared. Eight themes were evident—self-reported signs of psychological distress—anxiety, depression, difficulty coping, isolation; indicated that the health-care professional explaining their genetic results displayed limited knowledge of the condition and/or prognosis; expressed that waiting for the results of the genetic testing or clarity around the diagnosis had a negative impact on their decision-making in respect to their personal lives, i.e. family planning, education, employment; highlighted concerns about the lack of timely access to follow-up care or treatment; indicated an impact on wider relatives, indicated dissatisfaction at the process of service provision, e.g. consent, time frame, communication; expressed ongoing uncertainty around the diagnosis, prognosis or the wider implications. A number of responses also indicated satisfaction with their experience of accessing genetic testing and clinical genetic services. The number of quotes pertaining to each theme is outlined in Table 4.

**Table 4:** Emerging themes from free-text quotes

Theme		n	Quote example
1	Self-reported signs of psychological distress – anxiety, depression, difficulty coping, isolation	26	‘This is very frustrating and causing a lot of stress and anxiety for us as we have no clue of how this deletion will impact on us if we chose to get pregnant again.’ ‘A frustrating time….fear of the unknown, worry for the future. Scariest time of my life.’
2	Indicated that the health care professional explaining their genetic results displayed limited knowledge of the condition and/or prognosis	8	‘We need understanding and don’t have it. Someone to go through the report as can’t afford to go aboard.’ ‘Our paeds knew nothing about our son’s condition.’ ‘Yes, but their knowledge was admittedly very limited.’ ‘Gave results but did not satisfactorily explain.’
3	Expressed that waiting for the results of the genetic testing or clarity around the diagnosis had a negative impact on their decision-making with respect to their personal lives ie. family planning, education, employment	4	‘As my partner’s health was declining, the delay in my results gravely impacted our ability to think about starting a family…’ ‘18 months waiting and still not seen. Have put off having further children and it now may be too late’
4	Highlighted concerns about the lack of timely access to follow-up care or treatment	14	‘Delayed any medical interventions’ ‘Delayed plans to access possible treatment while my daughter’s sight continues to deteriorate’ ‘The length of time taken for results is too long and has resulted in delayed treatment.’
5	Indicated an impact on wider relatives	7	‘Impact on extended family. We are finished ours but not knowing if heredity or not places stress on extended family’ ‘The test and results took too long though. I worry now for my siblings, who are still waiting to even have the test. There are medical checks they’re not doing yet because they don’t know if they have the genetic mutation.’ ‘Extremely stressful time as my husband’s diagnosis has a profound effect on our adult children who are all of childbearing age.’ ‘We have six adult children and it has profound ramifications for them and our grandchildren’
6	Indicated dissatisfaction at the process of service provision eg. consent, time frame, communication	69	‘We were unaware that the consultant ran this genetic test when we attended A&E.’ ‘…didn’t know our son was being tested never told.’ ‘I did not understand consequences as I had no idea what Huntington’s was.’ ‘I got it by phone call but it was a very brief conversation and I was probably in the middle of cooking dinner when I got the call - was very difficult to process the result - I think I would have preferred an appointment for that call so I could prepare questions and to receive it via video. I also would have liked a letter to confirm the result - I have no written record of it.’
7	Expressed ongoing uncertainty around the diagnosis, prognosis or the wider implications	7	‘It is always on my mind whether or not it would be a good ethical decision for me to have children of my own in the future.’ ‘…we have no clue of how this deletion will impact on us if we chose to get pregnant again…’ ‘We were not given a definite timeframe, instead it was a rollercoaster of appointments and a lot of varying information.’
8	Indicated satisfaction with their experience of accessing genetic testing and Clinical Genetic services	12	‘Consultant was wonderful, an expert, very knowledgeable’ ‘My experience was good, the counsellor was great, really helpful.’ ‘I found it very supportive, it was arranged from there for other members of my family to be screened if they wished, they had the information that otherwise would never have known.’

### Impact of the waiting list

Fifty-six per cent of people sought out ways to access genetic testing or clinical genetic services while on the waiting list (Table 5). Respondents accessed genetic testing predominantly via public services (33.3%) mainly through a non-genetic consultant (31.9%, 44/138). Notably, 82% (116/142) reported a negative impact on their personal life due to the waiting list. Adverse effects on both personal and social choices with 59.9% (85/142) reporting delayed reproductive choices or plans to commit to a relationship or tension in personal relationships and 14.8% (21/142) delayed or changed plans in relation to education, employment, insurance. Of note, 23% (33/142) indicated a wider impact on the personal life and plans of extended family members (Table 5).

**Table 5:** Impact of the waiting list on patients

		*N*	*%*
While waiting for genetic counselling in Ireland (*n*=138)	Had genetic testing via GP / family doctor	2	1.4%
	Genetic testing via public consultant	44	31.9%
	Private genetic testing in Ireland	9	6.5%
	Private genetic testing or appointment is unaffordable	3	2.2%
	Appointment to see a Genetic Consultant or Genetic Counsellor via the Cross-Border Directive or the Treatment Abroad Scheme	3	2.2%
	Genetic testing via research study / clinical trial	9	6.5%
Reported impact of waiting time on personal life (*n*=142)	Delayed plans to have more children	25	17.6%
	Delayed plan to marry/settle down/commit to a relationship	6	4.2%
	Delayed plans to start a family	9	6.3%
	Delayed plans for mortgage or insurance	2	1.4%
	Changed/delayed employment	8	6%
	Changed/delayed education	11	8%
	Placed tension on relationships with partner, family members or friends	45	32%
	Wider impact on relative’s family planning/ relationships/ education/ employment plans	33	23%

### Genetic counsellor role

**Fig. 2:**
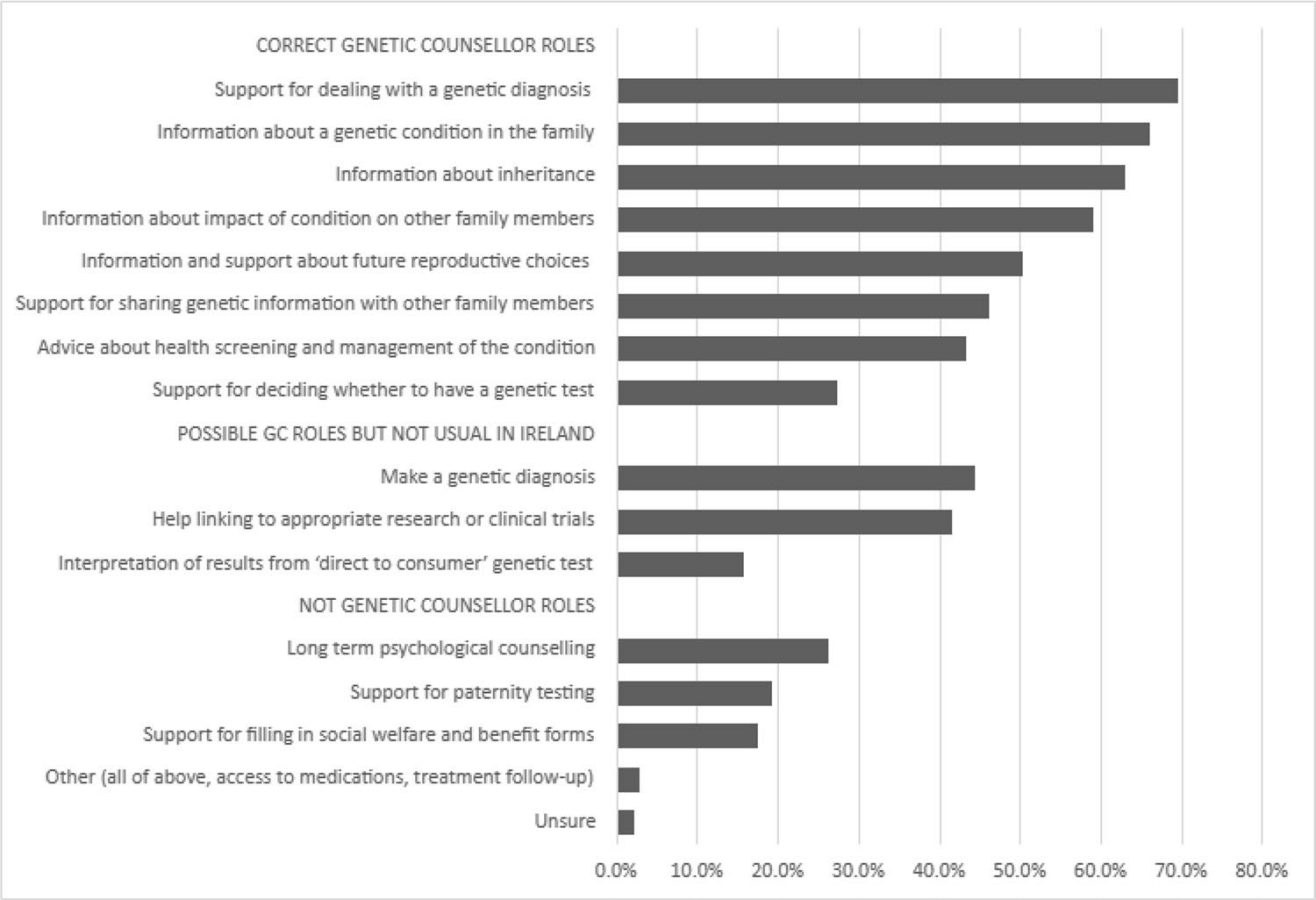
Participant (*n*=171) responses as to what they believe is included in the role of a genetic counsellor

In total, 171 participants responded to the question about the role of a genetic counsellor (Figure 2). While participants more frequently (65–79%) recognized the role of the genetic counsellor in providing information about a genetic condition in the family and support for dealing with a genetic diagnosis, fewer (28%) recognized the genetic counsellor’s role in supporting a decision whether to have genetic testing. Twenty-six per cent of participants erroneously felt that genetic counsellors provided long-term psychological counselling.

## Discussion

This survey aimed to capture the experiences of people with rare diseases and their families in accessing clinical genetic services in Ireland. Participants from all Irish community health regions were represented. In total, 36.2% lived in the Greater Dublin area in line with the census statistics (Central Statistics Office 2022) which indicate that this accounts for 39.2% of the population. Respondents reported a wide range of aspects of health affected by their rare condition reflecting participation from a diverse range of patients (Fig. 1a) and highlighting the multi-systemic nature of many genetic conditions (Fig. 1b). This requires multidisciplinary care involving numerous health-care professionals (HCPs) and places complex demands on healthcare systems, patients and families.

The power of genomics has been evidenced in undiagnosed cohorts (Wedderburn et al. 2021). A genetic diagnosis can have clinical implications for patients and ramifications for relatives—clinical genetics services play a key role in the patient pathway. Respondents reported a high diagnostic yield (71.5%), which is significantly higher than that expected using current optimal genetic testing methods. This suggests some bias in respondents being more likely those who had (or whose child) had received a diagnosis. As the survey was disseminated via the patient organization alliance Rare Diseases Ireland, which advocates for families with a rare disease diagnosis, this may account for any bias. Receiving a diagnosis through genetic testing or a clinical genetics appointment was associated with higher levels of satisfaction with the overall experience. We acknowledge that mainstreaming is necessary to ensure optimal patient care, particularly in situations where rapid diagnostics informs management decision-making. The development of standard operating procedures (SOPs) to aid non-genetic clinicians in taking consent and support with complex reports should improve the patient experience.

Evidence in this study suggests that patients experienced delayed access to clinical genetics assessment, testing and appropriate clinical interpretation. A significant proportion of respondents self-reported anxiety, worry, lack of coping and distress associated with uncertainty about the diagnosis and familial implications and indicated concern over delayed access to onward referrals, management, and potential treatment.

There was a strong theme of ongoing uncertainty in free text quotes. In the health literature, the period of uncertainty is recognized as a maximal stress period for patients (Grupe and Nitschke 2013). As well as delay, genomic results can themselves be complex and uncertain with patients reporting distress and worry about the future (Bartley et al. 2020). Access to genetic counselling expertise can contribute positively to a patient’s understanding and psychological management of uncertainty and complexity in results (Makhnoon et al. 2019).

Disclosure of genetic risk information to relatives is challenging for families. Comments relating to difficulties communicating risk to wider family members were noted particularly respondents wanting to inform adult children, siblings and more extended relatives about their risk but not having the clarity to do this. This was reflected in the quote, *‘The feeling of panic increases as people input, as you have no solid information for their questions it adds to the feeling of everything being out of control’* (Table 4). Communication of genetic risk with relatives outside the nuclear family can be more challenging than with closer relatives (Cody et al. 2008). Delays in reaching a diagnosis and uncertainty around implications for relatives can add complexity to the disclosure process for families (Studwell et al. 2021).

Responses indicating what actions participants had taken while waiting for a genetics appointment demonstrates the lack of options and cost barriers to finding a diagnosis by an alternate manner: while 33.3% had sought genetic testing from another health care provider, only 15.2% had accessed a genetic test via private testing, research studies or European Cross Border consultation. The lack of options on a lengthy clinical genetics waiting list no doubt contributes to the extensive personal impact: only 18% of respondents said there was no impact of being on the waiting list, and 32.0% (45/142) declared an impact on their personal relationships. The 17.6% (25/142) delaying plans to have a family is notable as Irish women are among the oldest in Europe to start a family with a mean age of 30.7 years (European Union 2021).

Our quantitative data highlight the ramifications for the wider family waiting for a genetic diagnosis in the family (Table 5).

Our survey shows evidence of significant levels of mainstream genetic testing activity by non-Genetics healthcare professionals with 76% (123/161) having their genetic testing arranged outside of the genetics clinic. A strong emergent theme was concerned that healthcare professionals delivering genetic results were not sufficiently knowledgeable to fully explain them. Of note, 25.0% (42/168) had a different team clinically interpret their genetic test results from those who arranged testing. Of those, 48.8% were referred to a genetics healthcare professional for full clinical interpretation with significant waiting periods (45% > 6 months) for an appointment. Non-genetic clinicians involved in the UK 100,000 Genomes project self-reported a lack of sufficient genetic knowledge to competently consent patients in, interpret and convey complex genomic testing results (Sanderson et al. 2019). Even genetics-trained HCPs report this as challenging (Vears et al. 2020). In 2019 the Genomics Education Programme from NHS Health Education England (Pichini and Bishop 2022) developed a cross-professional competency framework for consent and communication of germline genomic results to equip healthcare professionals in the delivery of genomic medicine in mainstream settings and initiatives to support genomic testing at the point of contact are ongoing (Copson et al. 2022). An Irish national genomic mainstreaming framework to upskill HCPs in appropriate genetic test selection, consent and clinical interpretation of genomic data would promote essential genomic literacy in this rapidly evolving field. For those clinicians who only order genetic tests occasionally, maintaining the skills necessary for optimum delivery of testing will be challenging, therefore education will need to be ongoing. Whilst opinions vary on the level of gatekeeping of genetic testing (Malgorzata et al. 2022) the legal framework implemented by European states and organizations strives to uphold the principles of informed consent, patient confidentiality, and the right to know and to refuse knowledge. A national genomic testing directory outlining testing repertoire with clinical criteria, test purpose, limitations, methodology, clinical validity, clinical utility and details of laboratory credentials would benefit service users. It has been suggested that a genetic test directory may, in certain cases, remove the need for referral to clinical genetics (Snape et al. 2019) and therefore may positively impact waiting times.

Survey responses suggest that patients value human interaction when receiving genetic testing results as in-person, telephone or video calls were preferred methods.

There is a need for public education about the availability of genetic counselling and the role of GCs. Misconceptions were evident, in particular expectations for the provision of long-term psychological counselling.

### Limitations of the study

A low number of male respondents 5.3% (9/171) participated in the survey and only 7% (12/171) of participants indicated they were from a non-Irish white background with no respondents from a Black, Asian, or mixed ethnic background. Figures from the Statistical Yearbook of Ireland (Central Statistics Office 2021) reported that 12.9% of people resident in Ireland were non-Irish nationals.

It was noted that the majority of the survey respondents were family members /

carers of a person living with a rare condition and answering on their behalf (67.8%, 116/171) and not those with a rare condition themselves (18.7%, 32/171). However, respondents did indicate that the person living with the rare condition had intellectual disability (43%), vision/eye problems (36.8%), behavioral difficulties (32.8%), and/or mental health challenges (21.6%) which may have impacted their ability to engage with the survey.

## Conclusion

Access to genetic testing and clinical genetic expertise is challenging in the current Irish healthcare system. Families report that long waits for clinical genetics services adversely impact well-being and curtail personal life plans for patients and wider family members. Investment in and improved access to clinical genetics expertise is a priority. This would ensure that families affected by rare genetic conditions have prompt access to healthcare professionals competent in clinical interpretation of complex genetic test results by clarifying recurrence risk and improving possible care and treatment prospects. However, the development of a national genetic test directory in conjunction with a genomic medicine competency framework may help guide non-genetic specialists to manage genetic test selection. This may allow less complex rare disease patients to be safely managed within a mainstream setting. We welcome the National Strategy for Accelerating Genetic and Genomic Medicine in Ireland and hope our findings will inform the effective implementation of the provision of national Clinical Genetic services.

### Supplementary information

ESM 1 (PDF 248 kb)

## Data Availability

The data that support the findings of this study are not openly available due to the personal nature of the information and the possibility of identification as respondents were families with rare diseases. These are available from the corresponding author upon reasonable request. Data are located in controlled access data storage at University College Dublin.

## Declarations

All procedures followed were in accordance with the ethical standards of the responsible committee on human experimentation (institutional and national) and with the Helsinki Declaration of 1975, as revised in 2000. Informed consent was obtained from all patients for being included in the study.

This article does not contain any studies with animal subjects performed by any of the authors.
