# Supplementary material for: Genetic services survey—experience of people with rare diseases and their families accessing genetic services in the Irish Republic
Source: J Community Genet. 2023 Aug 26;14(6):583–92. doi: 10.1007/s12687-023-00664-w (PMC10725380; doi:10.1007/s12687-023-00664-w)
Supplement: Supplementary file 1 — (PDF 248 kb) [file 12687_2023_664_MOESM1_ESM.pdf]

Rare Diseases Ireland

UCD RESEARCH

CHI Children's Health Ireland

Genetic Services survey

Genetic services survey January 2022: Information for Participants

We would like to find out more about the experience of adults and children when accessing genetic services in the Republic of Ireland, i.e., genetic testing and appointments with a Genetic Consultant or Genetic Counsellor. Please read this information to help you decide whether you would like to participate. You will also find this Information for Participants at rdi.ie/gxservices-survey/ if you would like to print a copy.

This survey is a co-operation between Rare Diseases Ireland (RDI - the national alliance for rare disease patient organisations in Ireland, www.rdi.ie) and a research team led by Prof. Sally Ann Lynch at University College Dublin/Children's Health Ireland (CHI) Crumlin, funded by the Adelaide Health Foundation. The title of the research project is "Genetic Counselling and Testing in the Irish Republic: Scoping current practice, international comparisons and recommendations for national practice".

What is the aim of this survey?
The aim of the survey is to gather experiences of people with rare diseases and undiagnosed conditions when accessing genetic services in Ireland. The findings will be used to recommend the future design of Irish genetic services. The survey should take about 15-20 minutes to complete.

Who can take part?
You can take part if you are aged 18 or over.
You can be:

- A person living with a rare condition – accessing genetic services for yourself and answering on your behalf
- A family member / carer of a person living with a rare condition accessing genetic services, and answering on their behalf
- A family member of a person living with a rare condition, accessing genetic services for yourself and answering on your behalf

Often in a family several people access genetic services. Each of these family members is welcome to complete the survey. A separate survey should be completed by each person who wishes to take part.

What questions will the survey ask?
The survey will first check that you consent to participate in this survey. There will then be questions about:

- Background information
- Experience of accessing a genetic test
- Experiences waiting for an appointment with a Genetic Consultant or Genetic Counsellor
- Experience of being seen by a Genetic Consultant or Genetic Counsellor
- Knowledge of genetic counselling

Who will know I have taken part?
We will not ask for your name, address, or birth date in the survey. Survey responses will be securely kept by Prof. Lynch at CHI Crumlin. Only RDI and the research team will have access to the survey responses, they won't be shared with anyone else.

Because we do not ask for names, we cannot identify your survey responses. This means if later you decide that you did not want to take part, we will not be able to withdraw your responses from the study.

How will survey responses be reported?
All survey responses will be combined so that no single response can be identified. RDI and the research team will analyse the results together. A report for policy makers, healthcare providers and the public will be published. We expect that a preliminary report will be available at Rare Diseases Day (February 28th) 2022. You will be able to access the final report from RDI (www.rdi.ie) after it has been published. The results of the analysis may also be reported in scientific journals and/or conferences.

Consenting to participate
It is up to you whether you choose to complete this survey. Please read this information carefully. The first survey questions will ask you if you are happy to consent to take part in the study. If you don't want to answer the survey, that's OK too.

As part of the consent, we ask if we may quote your text answers in the study report. You may choose not to have your quotes used in the report. You can still fill out the survey if you choose 'no' – your answers are still helpful. If you say 'yes', we may use some quotes in the report. We will not use any diagnoses or any identifying information in the quotes. However, because some situations are so rare, it is not possible to guarantee that you will not be identified from the quotes.

Privacy
Please do not use your name or the names of family members or healthcare professionals in your answers.

Who is funding the survey?
This survey work is funded by Adelaide Health Foundation (www.adelaide.ie), a voluntary independent charity which seeks to advance equal access to quality healthcare. Financial support for RDI's work is provided by several industry partners who have an interest in rare conditions. No funders have any role or influence in design, analysis or reporting of this research.

Potential Risks and Benefits
It is very unlikely that you would have any harm from completing this survey. You won't benefit directly by completing this survey. Findings from this study may help to influence policy makers to develop genetic services in Ireland.

If you have questions
If you have any general questions about this survey, please contact RDI at advocacy@rdi.ie. The RDI team can also help you fill in the survey if you are not comfortable completing an online survey yourself.

If taking part in this survey has raised any issues or concerns for you, please contact the research team at GCinIreland@gmail.com. They can answer your query or point you to appropriate supports or clinical services.

This information sheet and survey has been approved by the Children's Health Ireland Research Ethics Committee and Data Protection Office in June 2021. If you have concerns about data collected or stored during this study, please contact Prof. Sally Ann Lynch at GCinIreland@gmail.com, or the Data protection Office, CHI Crumlin at dataprotection@olchc.ie .

Data Protection Notice
All data will be collected and stored in line with the Data Protection Act 2018. The data will be kept on a secure encrypted device by Prof. Sally Ann Lynch. This data will be stored for five years in line with data protection guidelines and will be destroyed afterwards.

CHI Privacy Statement
CHI understands that your privacy is important to you and that you care about how your personal data is used. We respect and value the privacy of all of our research participants and will only collect and use personal data in ways that are described here, and in a way that is consistent with our obligations and your rights under the GDPR.

Read all the information about the CHI privacy statement for research participants here: privacy-notice-research-participant.pdf (olchc.ie)

Genetic Services survey

Section 1: About you – the person filling in the survey

Please do not use your name or the names of family members or healthcare professionals in your answers.

* 1. Are you aged 18 or over? You must be aged 18 or over to take part in this survey

- Yes
- No - survey will be exited

* 2. I consent to the data being used and stored as described in the information for participants

- Yes
- No - survey will be exited

3. I consent to my quotes being used in future publications about the survey findings as described in the information for participants

- Yes
- No

4. What is your gender?

- Male
- Female
- Other
- Prefer not to say

5. What is your ethnicity?

- Irish
- Irish Traveller
- Any other White background
- African
- Any other Black background
- Chinese
- Any other Asian background
- Other including mixed background
- Prefer not to say

6. Which best describes you?

- ◯ A person living with a rare condition accessing genetic services for myself and answering on my behalf
- ◯ A family member / carer of a person living with a rare condition accessing genetic services, and answering on their behalf
- ◯ A family member of a person living with a rare condition, accessing genetic services for myself and answering on my behalf
- ◯ Prefer not to say

Genetic Services survey

About the person accessing genetic services

Please tell us about the person who is accessing genetic services, and general information about the genetic condition. Please don't give us the precise genetic diagnosis

7. Where in the country do you / they live?

- CHO Area 1 - Cavan, Donegal, Leitrim, Monaghan, Sligo
- CHO Area 2 - Galway, Mayo, Roscommon
- CHO Area 3 - Clare, Limerick, Tipperary North
- CHO Area 4 - Cork, Kerry
- CHO Area 5 - Carlow, Kilkenny, Tipperary South, Waterford, Wexford
- CHO Area 6 - Dublin South East, Dun Laoghaire, Wicklow
- CHO Area 7 - Dublin South, Kildare, West Wicklow
- CHO Area 8 - Laois, Longford, Louth, Meath, Offaly, Westmeath
- CHO Area 9 - Dublin North
- Northern Ireland

>

- Other (please specify)

8. What age are you / they?

- Less than 1 year old
- 1-5 years old
- 6-15 years old
- 16-25 years old
- 26-50 years old
- 50+ years old

9. Which aspects of health are affected by the condition? (Tick all that apply)

- [ ] Heart (Cardiology)
- [ ] Breathing/lungs (Respiratory)
- [ ] Hormones/diabetes, (Endocrinology)
- [ ] Brain/nerves/spinal cord (Neurology & Neuromuscular)
- [ ] Muscles/ligaments/joints/inflammatory (Rheumatology)
- [ ] Bones/joints (Orthopaedics)
- [ ] Skin (Dermatology)
- [ ] Stomach/Digestion/Liver (Gastroenterology)
- [ ] Kidneys (Nephrology)
- [ ] Cancer (Oncology)
- [ ] Blood (Haematology)
- [ ] Reproductive
- [ ] Metabolic Diseases
- [ ] Vision/Eye (Ophthalmology)
- [ ] Hearing (Auditory)
- [ ] Mental Health (Psychiatry)
- [ ] Behavioural difficulties
- [ ] Intellectual disabilities
- [ ] Urological (uro-recto-genital)
- [ ] Craniofacial & ENT
- [ ] Birth defects / Congenital malformations
- [ ] Immune system (Immunology)

>

- [ ] Other (please specify)

10. I am seeking access to Genetic services because of a named condition?

- ( ) Yes
- ( ) No – still seeking a diagnosis
- ( ) No - Syndrome Without A Name (SWAN)
- ( ) Unsure

>

- ( ) Other (please specify)

Genetic Services survey

Accessing genetic testing

11. Have you / they had genetic testing?

- ◯ Yes
- ◯ No
- ◯ Unsure

>

- ◯ Other (please specify)

12. If yes, who arranged the genetic test?

- ◯ Genetic Consultant
- ◯ Genetic Counsellor
- ◯ GP / family doctor
- ◯ Unsure
- ◯ None of the above

>

- ◯ Another consultant (please add type of consultant below, for example Cardiologist)

13. Did the same person or team who arranged the genetic test explain the test result to you / them?

- ◯ Yes
- ◯ Unsure
- ◯ No - please tell us more in the next question

>

- ◯ Other (please specify)

14. If the person or team who ordered the genetic test did not give you the genetic test result, which type of professional gave you the results?

- ◯ Genetic consultant
- ◯ Genetic counsellor
- ◯ GP or family doctor
- ◯ Unsure

>

- ◯ Another consultant (for example Cardiologist or Opthalmologist), please specify

15. How long did you/they wait, after your/their sample was taken, for the genetic test result?

- ◯ 0-3 months
- ◯ 4-6 months
- ◯ 7-12 months
- ◯ 13-18 months
- ◯ More than 18 months
- ◯ Unsure
- ◯ Still waiting

16. How did you/they receive the genetic test result? (Tick all that apply)

- ☐ In-person appointment
- ☐ Phone call
- ☐ Letter
- ☐ E-mail
- ☐ Video call
- ☐ Parent/guardian told me/them the result
- ☐ Another family member told me/them the result
- ☐ Still waiting

>

- ☐ Other (please specify)

17. Did the genetic test give you/them a diagnosis?

- Yes
- No
- Unsure

>

- Other (please specify)

18. How would you/they have preferred to receive the genetic test result? (Tick all that apply)

- In-person appointment
- Phone call
- Letter
- E-mail
- Video call

>

- Other (please specify)

19. How satisfied are you/they with the overall experience of genetic testing?

- Very satisfied
- Quite satisfied
- Neither satisfied nor dissatisfied
- Quite dissatisfied
- Very dissatisfied
- Not applicable

Genetic Services survey

Access to Genetic Consultants and Genetic Counsellors

20. Have you/they been referred to see a Genetic Consultant or Genetic Counsellor?

- ◯ Yes - have already attended
- ◯ Yes - waiting for first appointment
- ◯ No - have not been referred, but would like to be
- ◯ No - have not been referred and do not want to be
- ◯ Unsure

>

- ◯ Other (please specify)

[ ]

21. In which hospital were you/they seen by a Genetic Consultant or Genetic Counsellor? Please only add the hospital name and do NOT name the healthcare professionals

[ ]

22. Was/is the Genetic Consultant or Genetic Counsellor appointment

- ◯ Private
- ◯ Public
- ◯ Provided by the ‘direct to consumer’ over the counter/ internet/ mail test kit company
- ◯ Prefer not to say
- ◯ Not applicable – I didn’t have an appointment

23. How long did you/they wait for the appointment with a Genetic Consultant or Genetic Counsellor? (If you/they are still waiting for an appointment - how long have you/they been waiting for?)

- ◯ 0-3 months
- ◯ 3-6 months
- ◯ 6-9 months
- ◯ 9-12 months
- ◯ 12-15 months
- ◯ 15-18 months
- ◯ 18-24 months
- ◯ More than 2 years
- ◯ Unsure
- ◯ Not applicable

24. While waiting to see a Genetic Consultant or Genetic Counsellor in Ireland I/they had: (Tick all that apply)

- ☐ genetic testing via GP
- ☐ genetic testing via public Consultant
- ☐ private genetic testing in Ireland
- ☐ private genetic testing or appointment is not something I/they could afford
- ☐ appointment to see a Genetic Consultant or Genetic Counsellor via the Cross-Border Directive or the Treatment Abroad Scheme
- ☐ genetic testing through a research study / clinical trial
- ☐ genetic testing via ‘direct to consumer’ over the counter/ internet/ mail test kits (e.g. 23 and me, Ancestry DNA, etc.)
- ☐ unsure
- ☐ none of the above

>

- ☐ private genetic testing in another country - name of country

25. What impact did being on the waiting list have on your personal life or plans? (Tick all that apply)

- ☐ delayed plans to have more children
- ☐ delayed plans to marry/settle down/commit to a relationship
- ☐ delayed plans to start a family
- ☐ delayed plans for mortgage or insurance
- ☐ changed or delayed employment
- ☐ changed or delayed education
- ☐ placed tension on relationships with partner, family members or friends
- ☐ wider impact on relative’s family planning/relationships/education/employment plans
- ☐ no impact
- ☐ not applicable

>

- ☐ Other (please specify)

26. We would like to hear more about your/their experience of being on the waiting list to see a Genetic Consultant or Genetic Counsellor. Please tell us about what could have been improved, what worked well and what didn’t work well.

27. Did the appointment with a Genetic Consultant or Genetic Counsellor give you/them a diagnosis?

- ◯ Yes
- ◯ No
- ◯ Unsure

>

- ◯ Other (please specify)

28. How satisfied are you/they with the overall experience of an appointment with a Genetic Consultant or Genetic Counsellor?

- ◯ Very satisfied
- ◯ Quite satisfied
- ◯ Neither satisfied nor dissatisfied
- ◯ Quite dissatisfied
- ◯ Very dissatisfied
- ◯ Not applicable

Genetic Services survey

Final Questions

29. Whether you have met with a genetic counsellor or not, what do you believe genetic counselling can provide? (Tick all that apply)

- [ ] Support for dealing with a genetic diagnosis in the family
- [ ] Long term psychological counselling
- [ ] Information about a genetic condition in the family
- [ ] Support for paternity testing
- [ ] Make a genetic diagnosis
- [ ] Information about how the condition is passed on (inherited) in the family
- [ ] Interpretation of genetic testing results from 'direct to consumer' over the counter/ internet/ mail tests (e.g. 23 and me, Ancestry DNA, etc.)
- [ ] Support for deciding whether to have a genetic test
- [ ] Support for sharing genetic information with other family members
- [ ] Support for filling in social welfare and benefit forms
- [ ] Advice about health screening and management of the condition
- [ ] Information about how a genetic condition may affect other family members
- [ ] Help linking to appropriate research or clinical trials
- [ ] Information and support about reproductive choices for a future pregnancy
- [ ] Unsure

>

- [ ] Other (please specify)

30. Is there anything else you/they would like to say about the experience of genetic testing and / or appointment with a Genetic Consultant or Genetic Counsellor? Please do NOT name the healthcare professionals involved

Genetic Services survey

Survey Finished

Thank-you very much for completing this survey. If taking part in this survey has raised any issues or concerns for you, please contact the research team at GCinIreland@gmail.com. They can answer your query or point you to appropriate supports or clinical services.

A report for policy makers, healthcare providers and the public will be published. We expect that a preliminary report will be available at Rare Diseases Day (February 28th) 2022. You will be able to access the report at www.rdi.ie when the final report has been published.
